# Feasibility of low-dose radiotherapy for patients with stage I/II extranodal NK-/*T*-cell lymphoma, nasal type achieving complete response after l-asparaginase-containing chemotherapy

**DOI:** 10.1016/j.ctro.2022.10.014

**Published:** 2022-11-03

**Authors:** Jae Sik Kim, Noorie Choi, Il Han Kim, Tae Min Kim, Yoon Kyung Jeon, Ji Hyun Chang

**Affiliations:** a Department of Radiation Oncology, Seoul National University College of Medicine, Seoul, Republic of Korea; b Department of Radiation Oncology, Soonchunhyang University Seoul Hospital, Seoul, Republic of Korea; c Department of Radiation Oncology, Veterans Health Service Medical Center, Seoul, Republic of Korea; d Department of Internal Medicine, Seoul National University Hospital, Seoul, Republic of Korea; e Seoul National University Cancer Research Institute, Seoul National University College of Medicine, Seoul, Republic of Korea; f Department of Pathology, Seoul National University Hospital, Seoul, Republic of Korea; g Department of Radiation Oncology, Seoul National University Hospital, Seoul, Republic of Korea

**Keywords:** Extranodal NK-/*T*-cell lymphoma, Nasal type, l-asparaginase, Radiotherapy, Complete response

## Abstract

**Purpose:**

To assess treatment outcomes in patients with stage I/II extranodal NK-/*T*-cell lymphoma, nasal type (ENKTCL-NT) and the feasibility of low-dose radiotherapy (RT) for achieving complete response (CR, defined as showing no residual hypermetabolic uptake on positron emission tomography [PET] or no residual lesions on computed tomography [CT]) after l-asparaginase-containing chemotherapy (l-ASP).

**Materials and methods:**

Between 1992 and 2018, 76 patients with early-stage ENKTCL-NT who achieved CR or partial response (PR) after induction chemotherapy received adjuvant RT. RT doses (using biologically equivalent doses in 2 Gy fractions [EQD2]) and rates of local recurrence-free survival (LRFS), locoregional recurrence-free survival (LRRFS), distant metastasis-free survival (DMFS), progression-free survival (PFS), and cancer-specific survival (CSS) were determined.

**Results:**

Median follow-up was 5.1 years (range, 0.5–20.8). The median RT dose was 45 Gy (range, 20–54). The 5-year LRFS, LRRFS, DMFS, PFS, and CSS rates were 82.7 %, 78.2 %, 81.1 %, 68.7 %, and 84.4 %, respectively. CR after induction chemotherapy was notably linked to better survival outcomes across each endpoint. Survival outcomes were not affected either by the administration of l-ASP or EQD2 < 40 Gy in patients displaying CR after l-ASP. Adverse events (AEs) ≥ Grade 2 were significantly reduced with EQD2 < 40 Gy, compared with EQD2 ≥ 40 Gy.

**Conclusion:**

Achieving CR after chemotherapy was the most predictive factor of survival outcomes in early-stage ENKTCL-NT. Decreasing RT doses in patients with CR after l-ASP appeared to minimize the occurrence of AE without compromising LRR risk; however, longer follow-ups and cautious application are warranted.

## Highlights

• Feasibility of low-dose RT in patients who achieve CR to l-ASP-based chemotherapy.
• l-ASP or EQD2 < 40 Gy was not associated with all survival outcomes.
• RT dose de-escalation could be provided to decrease the adverse event.

## 1 Introduction

The standard treatment for patients with extranodal NK-/*T*-cell lymphoma, nasal type (ENKTCL-NT) includes a combination of radiotherapy (RT; ≥ 50 Gy) and chemotherapy [1], [2]. In chemotherapy, conventional cyclophosphamide, vincristine, doxorubicin, and dexamethasone (CHOP) regimen has been used. However, because ENKTCL-NT cells may express multidrug resistance genes, the survival outcome is unfavorable as the rate of 3-year progression-free survival (PFS) is only 42.9 % with chemotherapy [3], [4].

The use of l-asparaginase-based chemotherapy (l-ASP) has been reported to improve disease-free and overall survival (OS) rates, and the complete response (CR) rate with CHOP regimen has been demonstrated to increase from 20 % to 72.5 % to 81.6 %–90.2 % during early ENKTCL-NT [5], [6], [7], [8]. In a retrospective study by Wang et al., following chemotherapy, a reduced RT dose of ≤50 Gy was administered to patients after they achieved CR, and no differences in PFS and OS rates were observed according to the RT dose [9].

The recommended target volume for ENKTCL-NT is typically larger than that for other lymphomas. Moreover, as most of the ENKTCL-NT develop in the nasal cavity, neighboring sites, such as the optic nerve and retina, are frequently at risk of damage because of their proximity to the target [10]. Therefore, in this study, we evaluated the outcomes for patients with stage I/II ENKTCL-NT and determined the feasibility of administering lower doses of RT in patients showing CR or partial response (PR) post l-ASP.

## 2 Materials and methods

### 2.1 Participants

Medical records of patients who were pathologically diagnosed with stage I or II ENKTCL-NT between January 1992 and December 2018 were reviewed. Patients who were treated with sequential chemotherapy and adjuvant RT and had undergone interim response assessment between chemotherapy and radiotherapy using 18F-fluoro-deoxy-glucose (FDG) positron emission tomography (PET) or computed tomography (CT) were included in the study. However, those with stable or progressive disease upon interim assessment were excluded.

In accordance with the scheme of a phase II trial on concurrent chemoradiotherapy for patients with ENKTCL-NT showing CR or PR following l-ASP [11], our institution has reduced the dose of adjuvant radiation for patients showing CR or PR post l-ASP since 2013. Moreover, we compared various dose fractionations using an equivalent dose in 2 Gy fractions (EQD2, α / β = 10). CR was defined as no residual hypermetabolic uptake in the tumor corresponding to a Deauville score of 1 or 2 on PET scan or no residual tumor on CT scan. PR was defined as regressed standardized uptake value on PET scan or a smaller tumor on CT scan according to the Lugano classification [12].

### 2.2 Ethics Statement

This study was approved by the Institutional Review Board of Seoul National University Hospital (approval number: H-1505-042-670), which waived the requirement for obtaining informed consent from participants because of the retrospective design of the study. All procedures in this study involving human participants were performed in accordance with the ethical standards of the institutional and/or national research committee as well as the 1964 Helsinki declaration and its later amendments or comparable ethical standards.

### 2.3 Adverse events

We reviewed all adverse events (AEs) occurring at adjacent tissues to the nasal cavity: mucositis, dermatitis, dry nose and mouth, oral pain, epistaxis, nasal obstruction, and others. These were graded by Common Terminology Criteria for Adverse Events v5.0.

### 2.4 Statistical analysis

Local recurrence-, locoregional recurrence-, and distant metastasis-free survival (LRFS, LRRFS, and DMFS, respectively) were defined as the interval from the diagnosis to the local, locoregional, or distant failure, respectively. PFS was calculated from the date of diagnosis to the date of disease progression or death and cancer-specific survival (CSS) was to the date of cancer-related death. These survival outcomes were calculated using the Kaplan–Meier method, and they were compared using the log-rank test. The primary endpoint of the present study was LRRFS.

The Cox proportional-hazard model was used for survival analyses. Multivariate analysis used covariates with p-values of < 0.15 in univariate analyses. A two-tailed p-value of < 0.05 was considered to be statistically significant. All the data analyses were conducted using Stata version 16 (StataCorp, College Station, TX).

## 3 Results

Overall, 97 consecutive patients with stage I/II ENKTCL-NT received chemotherapy between January 1992 and December 2018. The results of the study revealed that CR was achieved in 33 (55 %) patients who were not treated with l-ASP and 27 (73 %) patients who were with l-ASP. PET and CT scans were more frequently used to determine CR in the l-ASP group than in the non-l-ASP group (n = 22, 81 % vs n = 7, 21 %; p < 0.001). In addition, a significant correlation was observed between l-ASP and increased CR or PR after chemotherapy (p = 0.003), and the correlation was maintained after adjusting for the covariates of gender, age, stage, and B symptoms. Of the 97 patients, 8 who did not receive RT after CR and 13 who had progressive disease were excluded from the analyses. The remaining 76 patients who received RT and whose 18F-FDG PET or CT scan revealed CR or PR after chemotherapy were included in the analyses.

Of these 76 patients, 40 were included in the non-l-ASP group. In total, 32 patients received combination therapy of ifosfamide, etoposide, methotrexate, and prednisolone [IMEP] or ifosfamide, methotrexate, VP-16, and prednisone [IMVP-16/Pd], and 8 patients received CHOP regimen. The remaining 36 patients were included in the l-ASP group, i.e., they received IMEP and l-ASP. IMEP was initially administered in six cycles; however, because of early local failures, it was reduced to three cycles. In 17 patients, l-ASP was administered in the pegylated form (ONCASPAR, pegaspargase). Regarding the RT scheme, the median dose of 45 Gy was administered in 25 fractions (range, 20 Gy/4 fractions–54 Gy/30 fractions). Only one patient received 20 Gy in 4 fractions because of the spatial accessibility to the hospital. When patients were classified into two groups according to EQD2 of < 40 Gy, 4 patients in the non-l-ASP and 19 patients in the l-ASP group received EQD2 of < 40 Gy (p < 0.001). These four patients in the non-l-ASP group with EQD2 < 40 Gy had undergone incomplete RT because of AE; however, they were included in analyses for the comparisons based on AE outcomes. As we had intentionally reduced the dose of adjuvant radiation in patients showing CR or PR post l-ASP, patients with CR accounted for 95.7 % and 56.6 % of patients treated with EQD2 of < 40 Gy and ≥ 40 Gy, respectively (p < 0.001). Table 1 shows the patient characteristics.

**Table 1:** Patient and tumor characteristics based on chemotherapy regimens with or without l-asparaginase.

		**Non-l-ASP**	**l-ASP**	**p-value**
		**N = 40**	**N = 36**	
		**N (%)**	**N (%)**	
Gender	Male	24 (60.0 %)	22 (61.1 %)	1.00
	Female	16 (40.0 %)	14 (38.9 %)	
Age	< 60 years	29 (72.5 %)	24 (66.7 %)	0.62
	≥ 60 years	11 (27.5 %)	12 (33.3 %)	
Ann Arbor stage	I	30 (75.0 %)	23 (63.9 %)	0.33
	II	10 (25.0 %)	13 (36.1 %)	
B symptoms	0	31 (77.5 %)	28 (77.8 %)	1.00
	1	9 (22.5 %)	8 (22.2 %)	
Initial LDH	≤ 225 IU/L	22 (55.0 %)	21 (58.3 %)	1.00
	> 225 IU/L	15 (37.5 %)	14 (38.9 %)	
	Missing	3 (7.5 %)	1 (2.8 %)	
RT dose	EQD2 ≥ 40 Gy	36 (90.0 %)	17 (47.2 %)	< 0.001
	EQD2 < 40 Gy	4 (10.0 %)	19 (52.8 %)	
Response after CTx	CR	25 (62.5 %)	27 (75.0 %)	0.32
	PR	15 (37.5 %)	9 (25.0 %)	
RT technique	3D-CRT	34 (85.0 %)	15 (41.7 %)	< 0.001
	IMRT	6 (15.0 %)	21 (58.3 %)	

l-ASP, l-asparaginase-containing chemotherapy; LDH, lactate dehydrogenase; RT, radiotherapy; EQD2, equivalent dose in 2 Gy fractions; CTx, chemotherapy; CR, complete response; PR, partial response; 3D-CRT, three-dimensional conformal radiation therapy; IMRT, intensity-modulated radiation therapy.

The median follow-up time for this cohort was 5.1 years (range, 0.5–20.8), with patients in the non-l-ASP and l-ASP groups being followed up for 7 and 4.5 years, respectively. The 5-year LRFS, LRRFS, DMFS, PFS, and CSS rates were 82.7 %, 78.2 %, 81.1 %, 68.7 %, and 84.4 %, respectively. Interestingly, even after adjusting for other variables, CR following initial chemotherapy was associated with better outcomes in each endpoint—LRFS (p = 0.033), LRRFS (p = 0.023), DMFS (p = 0.041), PFS (p = 0.003), and CSS (p = 0.013). Furthermore, the effects of l-ASP and EQD2 ≥ 40 Gy administration on survival outcomes were comparable. In a subgroup analysis of patients who achieved CR post l-ASP (n = 27), none of the variables (including RT dose) were related to survival endpoints, and the 5-year LRFS, LRRFS, DMFS, PFS, and CSS in this subgroup were 87.1 %, 87.1 %, 96.1 %, 83.2 %, and 100 %, respectively. Of the 27 patients, 18 received low-dose RT (EQD2 < 40 Gy). Results of the survival analyses are shown in Table 2.

**Table 2:** Univariate and multivariate Cox regression analyses for treatment outcomes.

Variables	Local recurrence-free survival				Locoregional recurrence-free survival				Distant metastasis-free survival				Progression-free survival				Cancer-specific survival			
	Univariate		Multivariate		Univariate		Multivariate		Univariate		Multivariate		Univariate		Multivariate		Univariate		Multivariate	
	P value	HR (95 % CI)	P value	HR (95 % CI)	P value	HR (95 % CI)	P value	HR (95 % CI)	P value	HR (95 % CI)	P value	HR (95 % CI)	P value	HR (95 % CI)	P value	HR (95 % CI)	P value	HR (95 % CI)	P value	HR (95 % CI)
age ≥ 60 years	0.076	2.69 (0.90–8.00)	0.028	3.76 (1.15–12.20)	0.252				0.711				0.943				0.994			
l-ASP	0.195				0.591				0.355				0.626				0.307			
Female	0.890				0.564				0.428				0.418				0.920			
stage II	0.103	2.48 (0.83–7.40)	0.040	3.43 (1.05–11.11)	0.035	2.88 (1.08–7.67)	0.048	2.70 (1.01–7.22)	0.286				0.046	2.24 (1.01–4.93)	0.061	2.13 (0.96–4.71)	0.143	2.26 (0.76–6.74)	0.090	3.29 (0.83–12.99)
B symptoms	0.255				0.409				0.187				0.655				0.105	2.52 (0.82–7.73)	0.030	5.12 (1.18–22.36)
EQD2 < 40 Gy	0.439				0.841				0.082	0.17 (0.02–1.26)	0.965		0.259				0.901			
LDH_preCTx_ > 225 IU/L	0.189				0.249				0.745				0.623				0.379			
LDH_postCTx_ > 225 IU/L	0.726				0.938				0.068	3.14 (0.92–10.75)	0.205		0.205				0.141	2.59 (0.73–9.17)	0.630	0.68 (0.14–3.28)
LDH_postRT>_225 IU/L	0.957				0.954				0.513				0.922				0.459			
CR after CTx	0.049	2.99 (1.00–8.92)	0.033	3.40 (1.10–10.46)	0.017	3.34 (1.24–8.97)	0.023	3.16 (1.17–8.51)	0.004	4.83 (1.65–14.15)	0.041	4.03 (1.06–15.29)	0.002	3.42 (1.55–7.55)	0.003	3.32 (1.51–7.33)	0.006	5.16 (1.59–16.76)	0.013	8.24 (1.57–43.36)
Use of IMRT	0.265				0.299				0.293				0.952				0.083	0.16 (0.02–1.27)	0.093	0.14 (0.01–1.39)

HR, hazard ratio; CI, confidence interval; l-ASP, l-asparaginase-containing chemotherapy, EQD2, equivalent dose in 2 Gy fractions; LDH_preCTx,_ lactate dehydrogenase before chemotherapy; LDH_postCTx,_ lactate dehydrogenase after chemotherapy; LDH_postRT,_ lactate dehydrogenase after radiotherapy; CR, complete response; CTx, chemotherapy; IMRT, intensity-modulated radiation therapy.

**Fig. 1:**
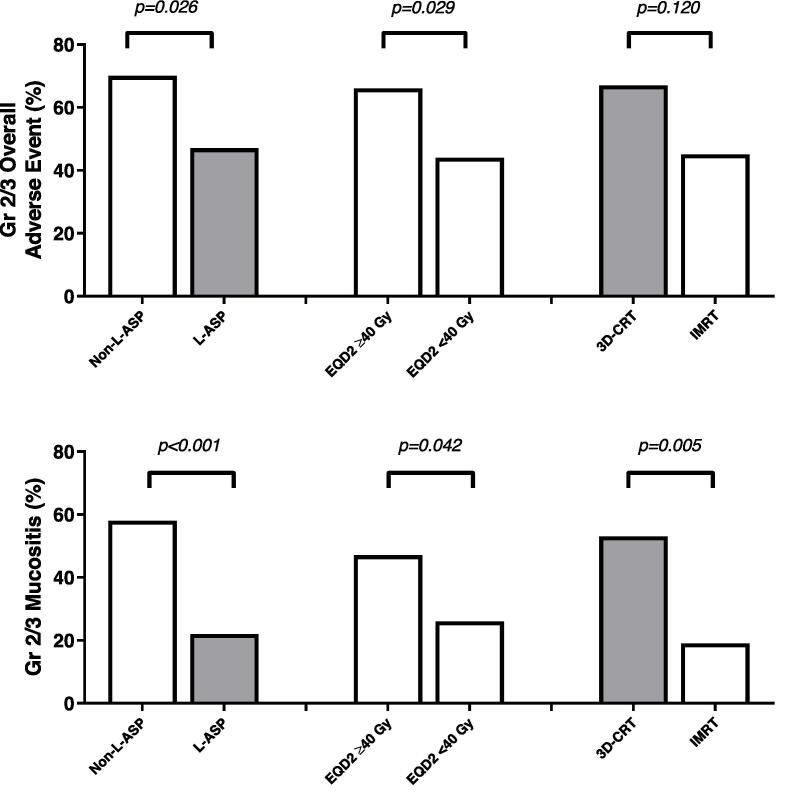
Adverse event profile after radiotherapy for patients with stage I/II extranodal NK-/*T*-cell lymphoma, nasal type. Gr, grade; l-ASP, l-asparaginase-containing chemotherapy; EQD2, equivalent dose in 2 Gy fractions; 3D-CRT, three-dimensional conformal radiation therapy; IMRT, intensity-modulated radiation therapy.

The use of l-ASP (p = 0.026) and EQD2 of < 40 Gy (p = 0.029) was associated with a lower incidence of AE of ≥ Grade 2 (Fig. 1). Further analysis of possible mucositis-predicting variables—use of l-ASP (p < 0.001), EQD2 < 40 Gy (p = 0.042), and intensity-modulated radiation therapy (p = 0.005)—revealed an association with a lower incidence of mucositis of ≥ Grade 2. Table 3 presents failure patterns after the treatment for stage I/II ENKTCL-NT according to the chemotherapy regimen and EQD2 of RT.

**Table 3:** Patterns of failure after treatment for stage I/II extranodal NK-/*T*-cell lymphoma, nasal type.

		N	F/U years (median, range)	Local failure (LF)	Regional failure (RF)	Distant failure (DF)
Non-l-ASP	EQD2 ≥ 40 Gy	36	5.2 (0.5–18.6)	4 (11 %)	3 (8 %) (without LF)	9 (25 %) (1 with LF, 1 with RF, 7 DF only)
	EQD2 < 40 Gy	4	8.4 (2.8–20.8)	1 (25 %)	0	1 (25 %) (with LF)
l-ASP	EQD2 ≥ 40 Gy	17	5.1 (0.6–11.3)	4 (24 %)	2 (12 %) (with LF)	5 (29 %) (1 with LF, 2 with locoregional, 2 DF only)
	EQD2 < 40 Gy	19	5.0 (0.9–9.4)	4 (21 %)	0	0

F/U, follow-up; l-ASP, l-asparaginase-containing chemotherapy; EQD2, equivalent dose in 2 Gy fractions.

In addition, among eight patients who were excluded from the main analysis because they had not received RT after CR, three (37.5 %) were alive without recurrence, two (25 %) experienced local recurrence, and three died due to noncancerous causes (sepsis and infarct).

## 4 Discussion

In the present study, we analyzed treatment responses and survival outcomes in patients with early-stage ENKTCL-NT by focusing on the feasibility of low-dose RT in patients showing CR or PR on PET or CT scan post l-ASP. Our findings indicated no differences in LRFS, LRRFS, DMFS, PFS, and CSS rates between the groups receiving EQD2 of ≥ 40 Gy and < 40 Gy. Moreover, high RT dose was found to be related to AE and mucositis of ≥ Grade 2.

The standard treatment for early ENKTCL-NT includes a combination of RT and chemotherapy; however, the effectiveness of this regimen is yet to be fully established because it is rare, and only a few randomized controlled trials on this regimen have been conducted. The outcomes following the administration of the classical CHOP regimen are reported to be poor; therefore, non-anthracycline-based regimens containing ifosfamide and methotrexate are preferred [2], and treatment with l-ASP in ENKTCL-NT has shown better response rates and survival outcomes than the conventional chemotherapy. In a Chinese comparative study between l-ASP and CHOP regimen, l-ASP improved survival outcomes in early-stage ENKTCL-NT [13]. Another Korean study reported an improvement in survival in stage III/IV patients after adding l-ASP to the regimen [8]. Our study shows similar results as treatment with l-ASP was associated with a significantly higher rate of CR or PR than non-l-ASP (73 % vs 55 %).

Regarding RT, doses of ≥50 Gy have been considered to be essential for the treatment of ENKTCL-NT [14]. As this RT practice was set in the era of anthracycline-based chemotherapy, the RT dose could now be reduced with the use of more effective types of chemotherapy. For patients with ENKTCL-NT, the location of the lymphoma might be associated with the incidence of severe mucositis or xerostomia after RT [15]; therefore, de-escalation of the doses could help decrease the number of potential AEs. In our study, although the EQD2 < 40 Gy group comprised of patients who received low-dose RT because of AEs, we observed a significant relationship between the RT dose and post-treatment AE, especially for mucositis.

RT administration for the treatment of ENKTCL-NT occurs either concurrently, sequentially, or in the middle of chemotherapy (sandwich). Treatment outcomes with reduced doses of RT have been reported in some studies using concurrent chemoradiation therapy. In a study by Oh et al. [16], 96.5 % patients showed CR after 40 Gy was concurrently administered with cisplatin and consolidative chemotherapy. In another phase II Korean trial, 82.1 % patients showed CR with a RT dose of 36–44 Gy, followed by methotrexate, etoposide, ifosfamide, dexamethasone, l-asparaginase (MIDLE) chemotherapy [11]. With respect to sequential combination for NK-*T*-cell lymphoma, Wang et al. examined patients receiving > 50 Gy (median, 56 Gy) and ≤ 50 Gy (median, 46 Gy) RT who showed CR after initial chemotherapy (including non-l-ASP and l-ASP administration in the upper aerodigestive tract), and they observed no differences in PFS or OS, although the reason why RT dose was reduced in a small subset of patients remains unclear [9]. Similarly, the results of the present study demonstrated that lower doses of RT for patients with CR post l-ASP chemotherapy are not associated with reduced survival outcomes.

RT doses of 30–36 Gy are recommended for patients diagnosed with other lymphomas (e.g., diffuse large B-cell lymphoma [DLBCL] or peripheral *T*-cell lymphoma) who show CR after first-line chemotherapy [10]. This is supported by the findings from a randomized trial conducted by the British National Lymphoma Investigation, which examined RT de-escalation in non-Hodgkin’s lymphoma [17], and several retrospective studies targeting DLBCL [18], [19]. Based on this strategy, to improve the patients’ quality of life and preserve treatment efficacy, we performed a risk-adapted reduction in RT dose to lower than that used by Wang et al. [9] for patients showing CR post l-ASP.

Similar to any study, this study has some limitations and strengths. Previously, Deng et al. [20] had reported that the use of < 50 Gy could be related to increased locoregional failure; however, they neither mentioned the reason for decreasing the RT dose, which was presumed to be due to intolerance or AE, nor they routinely performed the evaluations using PET-CT or CT. In our investigation, despite the small cohort, we routinely conducted PET-CT or CT examinations between the end of chemotherapy and initiation of RT to evaluate responses. We showed that prognoses were comparable among l-ASP patients receiving low or high doses. Furthermore, the current investigation only supports the feasibility of using a low-dose RT. Therefore, caution should be exercised in mentioning its definitive superiority. In addition, a few patients in our cohort showed CR post l-ASP, i.e., we could only perform a subgroup analysis. Notably, in our study, no patient who received l-ASP and showed CR experienced cancer-related death, suggesting that particular attention must be paid to local control. Randomized controlled trials are important to confirm therapeutic success, and an ongoing Chinese study (ChiCTR-TRC-14005218) monitoring 2-year LRFS in patients receiving high and low (< 50 Gy) dose RT who showed CR after pegaspargase-based chemotherapy will provide further insight. However, more randomized controlled trials are needed to elucidate the feasibility of lower RT doses after l-ASP.

In conclusion, our study showed that the administration of RT dose of < 40 Gy did not affect survival outcomes in patients with early-stage ENKTCL-NT who had achieved CR post l-ASP. In contrast, higher RT doses were positively associated with AEs, such as mucositis. Hence, longer follow-up studies and careful use of low-dose RT are necessary. Further investigations on the potential risk factors of poor prognosis in early-stage ENKTCL-NT are required for the proper selection of patients who may benefit from lower RT doses without compromising the likelihood of survival.

## Author contributions

NC, IK, and JC contributed to the conception and design of the study. NC and JC organized the database and performed the statistical analysis. JK and JC wrote the first draft of the manuscript. All authors have contributed to manuscript revision as well as have read and approved the submitted version.

## Funding

This research did not receive any specific grant from funding agencies in the public, commercial, or not-for-profit sectors.

## Declaration of Competing Interest

The authors declare that they have no known competing financial interests or personal relationships that could have appeared to influence the work reported in this paper.

## References

[1] Wang H., Li Y.-X., Wang W.-H., Jin J., Dai J.-R., Wang S.-L. (2012). Mild toxicity and favorable prognosis of high-dose and extended involved-field intensity-modulated radiotherapy for patients with early-stage nasal NK/T-cell lymphoma. Int J Radiat Oncol Biol Phys.

[2] Kim S.J., Yoon S.E., Kim W.S. (2018). Treatment of localized extranodal NK/T cell lymphoma, nasal type: a systematic review. J Hematol Oncol.

[3] Yamaguchi M., Kita K., Miwa H., Nishii K., Oka K., Ohno T. (1995). Frequent expression of P-glycoprotein/MDR1 by nasal T-cell lymphoma cells. Cancer.

[4] Noh Y.J., Ahn Y.C., Kim W.S., Ko Y.H. (2004). Sequential chemoradiotherapy for stage I/II nasal natural killer/T cell lymphoma. Rad Oncol J.

[5] Lin N., Song Y., Zheng W., Tu M., Xie Y., Wang X. (2013). A prospective phase II study of L-asparaginase- CHOP plus radiation in newly diagnosed extranodal NK/T-cell lymphoma, nasal type. J Hematol Oncol.

[6] Jiang M., Zhang L.i., Xie L.i., Zhang H., Jiang Y.u., Liu W.-P. (2017). A phase II prospective study of the “Sandwich” protocol, L-asparaginase, cisplatin, dexamethasone and etoposide chemotherapy combined with concurrent radiation and cisplatin, in newly diagnosed, I/II stage, nasal type, extranodal natural killer/T-cell lymphoma. Oncotarget.

[7] Bu S., Yuan F., Wei X., Yin Q., Li Y., Mi R. (2016). L-asparaginase-based regimen as a first-line treatment for newly diagnosed nasal type extranodal natural killer cell/T-cell lymphoma. Exp Ther Med.

[8] Kim M., Kim T.M., Kim K.H., Keam B., Lee S.-H., Kim D.-W. (2015). Ifosfamide, methotrexate, etoposide, and prednisolone (IMEP) plus l-asparaginase as a first-line therapy improves outcomes in stage III/IV NK/T cell-lymphoma, nasal type (NTCL). Ann Hematol.

[9] Wang L. (2016). Radiation dose reduction for patients with extranodal NK/T-cell lymphoma with complete response after initial induction chemotherapy. Onco Targets Ther.

[10] Yahalom J., Illidge T., Specht L., Hoppe R.T., Li Y.-X., Tsang R. (2015). Modern radiation therapy for extranodal lymphomas: field and dose guidelines from the International Lymphoma Radiation Oncology Group. I*nt J Radiat Oncol Biol Phys*.

[11] Yoon D.H., Kim S.J., Jeong S.H., Shin D.-Y., Bae S.H., Hong J. (2016). Phase II trial of concurrent chemoradiotherapy with L-asparaginase and MIDLE chemotherapy for newly diagnosed stage I/II extranodal NK/T-cell lymphoma, nasal type (CISL-1008). Oncotarget.

[12] Cheson B.D., Fisher R.I., Barrington S.F., Cavalli F., Schwartz L.H., Zucca E. (2014). Recommendations for initial evaluation, staging, and response assessment of Hodgkin and non-Hodgkin lymphoma: the Lugano classification. J Clin Oncol.

[13] Huang L., Yuan B., Wu H., Chu H., Liu Y., Wu S. (2017). Comparative study of L-asparaginase-based LOP regimen over CHOP regimen before radiotherapy for stage IIE extranodal nasal type NK/T cell lymphoma: A Study of 2 Centers. Clin Lymphoma Myeloma Leuk.

[14] Wu T., Yang Y., Zhu S.-Y., Shi M., Su H., Wang Y. (2018). Risk-adapted survival benefit of IMRT in early-stage NKTCL: a multicenter study from the China Lymphoma Collaborative Group. Blood Adv.

[15] Yamaguchi M., Miyazaki K. (2017). Current treatment approaches for NK/T-cell lymphoma. J Clin Exp Hematop.

[16] Oh D., Ahn Y.C., Kim S.J., Kim W.S., Ko Y.H. (2015). Concurrent chemoradiation therapy followed by consolidation chemotherapy for localized extranodal natural killer/T-cell lymphoma, nasal type. Int J Radiat Oncol Biol Phys.

[17] Lowry L., Smith P., Qian W., Falk S., Benstead K., Illidge T. (2011). Reduced dose radiotherapy for local control in non-Hodgkin lymphoma: a randomised phase III trial. Radiother Oncol.

[18] Lee J.W., Prosnitz L.R., Stefanovic A., Kelsey C.R. (2019). Are higher doses of consolidation radiation therapy necessary in diffuse large B-cell lymphoma involving osseous sites?. Adv Radiat Oncol.

[19] Dorth J.A., Prosnitz L.R., Broadwater G., Beaven A.W., Kelsey C.R. (2012). Radiotherapy dose-response analysis for diffuse large B-cell lymphoma with a complete response to chemotherapy. Radiat Oncol.

[20] Deng X.-W., Wu J.-X., Wu T., Zhu S.-Y., Shi M., Su H. (2018). Radiotherapy is essential after complete response to asparaginase-containing chemotherapy in early-stage extranodal nasal-type NK/T-cell lymphoma: A multicenter study from the China Lymphoma Collaborative Group (CLCG). Radiother Oncol.

